# Biosynthetic Gene Pyramiding Leads to Ascorbate Accumulation with Enhanced Oxidative Stress Tolerance in Tomato

**DOI:** 10.3390/ijms20071558

**Published:** 2019-03-28

**Authors:** Xiaojing Li, Jie Ye, Shoaib Munir, Tao Yang, Weifang Chen, Genzhong Liu, Wei Zheng, Yuyang Zhang

**Affiliations:** 1 Key Laboratory of Protected Horticultural Engineering in Northwest, College of Horticulture, Northwest Agriculture & Forestry University, Yangling, Shaanxi 712100, China; lixiaojing@nwafu.edu.cn; 2 Key Laboratory of Horticultural Plant Biology, Ministry of Education, Huazhong Agricultural University, Wuhan 430070, China; yejie@mail.hzau.edu.cn (J.Y.); bajwa82mna@gmail.com (S.M.); scyangtao@126.com (T.Y.); chenweifang@webmail.hzau.com (W.C.); liugenzhong@126.com (G.L.); 3 HZAU Chuwei Institute of Advanced Seeds, Wuhan 430070, China; cwsw2017@163.com

**Keywords:** Tomato (*Solanum lycopersicum*), AsA, gene pyramiding, biosynthesis pathway, oxidative stress

## Abstract

Ascorbic acid (AsA) has high antioxidant activities, and its biosynthesis has been well studied by engineering of a single structural gene (SG) in staple crops, such as tomato (*Solanum lycopersicum*). However, engineering the AsA metabolic pathway by multi-SG for biofortification remains unclear. In this study, pyramiding transgenic lines including GDP-Mannose 3′,5′-epimerase (*GME*) × GDP-d-mannose pyrophosphorylase (*GMP*), GDP-l-Gal phosphorylase (*GGP*) × l-Gal-1-P phosphatase (*GPP*) and *GME* × *GMP* × *GGP* × *GPP*, were obtained by hybridization of four key genes to get over-expression transgenic plants (*GME*, *GMP*, *GGP*, and *GPP*) in tomato. Pyramiding lines exhibited a significant increase in total ascorbate in leaves and red fruits except for *GGP* × *GPP*. Expression analysis indicated that increased accumulation of AsA in pyramiding transgenic lines is due to multigene regulation in AsA biosynthesis. Substrate feeding in leaf and fruit suggested that AsA biosynthesis was mainly contributed by the d-Man/l-Gal pathway in leaves, while alternative pathways may contribute to AsA accumulation in tomato fruit. Pyramiding lines showed an enhanced light response, stress tolerance, and AsA transport capacity. Also, fruit shape, fruit size, and soluble solids were slightly affected by pyramiding. This study provides the first comprehensive analysis of gene pyramiding for ascorbate biosynthesis in tomato. SGs pyramiding promotes AsA biosynthesis, which in turn enhances light response and oxidative stress tolerance. Also, the data revealed an alternative ascorbate biosynthesis pathway between leaves and fruit of tomato.

## 1. Introduction

l-Ascorbic acid (AsA, vitamin C) is a highly abundant and essential organic acid for plants and animals. As an antioxidant and enzyme cofactor, AsA plays a crucial role in various plant physiological processes, including removing reactive oxygen species (ROS) [1,2], enhancing oxidative stress tolerance [3], premature senescence and programmed cell death (PCD) [4,5], and cell elongation and division in plants [6]. It has been shown that 30% to 40% of AsA in plant cells is located in the chloroplast, which plants use to produce ROS under light respiration and photosynthesis, suggesting AsA plays a vital role in protecting against side effects of photosynthesis in plant [7,8]. In humans, AsA can scavenge free radicals, thereby preventing and suppressing the occurrence of cancer, reducing blood cholesterol, and enhancing the immune system [9,10]. Unfortunately, humans have lost the ability to synthesize vitamin C due to the functional loss of l-gulono-gamma-lactone oxidase [11]. Thus, fresh vegetables and fruits including tomato are considered as the primary sources of vitamin C in the human diet. 

Four pathways for biosynthesis of AsA have been identified (Appendix A1), including the d-mannose/l-galactose (d-Man/l-Gal) pathway, the d-gulose pathway, the d-galacturonate pathway, and the myo-inositol pathway [12,13,14,15]. The d-Man/l-Gal pathway is recognized as the most critical biosynthesis pathway in plants, and all structural genes in this pathway have been identified, including those encoding GDP-d-Man pyrophosphorylase (GMP) [16], GDP-Man-3′,5′-epimerase (GME) [13], GDP-l-Gal phosphorylase (GGP) [17], l-Gal-1-P phosphatase (GPP) [18], l-Gal dehydrogenase (GalDH) [19], and l-galactono-1,4-lactone dehydrogenase (GLDH) [20].

GMP is a rate-limiting enzyme in the d-Man/l-Gal pathway and is involved in the reversible conversion of d-mannose-1-phosphate to GDP-d-mannose. Several molecular studies have proved that *GMP* has an important role in the d-Man/l-Gal pathway. The AsA content in a *GMP* mutant named *vtc1* in Arabidopsis was 75% decreased compared to the wild-type. When *vtc1* plants were transformed with the wild-type VTC1 gene, foliar AsA concentration was restored to the wild-type level [21]. In the tomato genome, there are four members of *GMP* gene family (*GMP1*-*GMP4*). Previously, loss- and gain-of-function of *GMP3* in tomato resulted in a decreased and increased accumulation of AsA, respectively, confirming the fact that *GMP* plays a vital role in the biosynthesis of AsA in tomato [22,23]. GME, which is located downstream of GMP and converts GDP-d-mannose to GDP-l-galactose, is considered to be the central enzyme in AsA biosynthesis. Transgenic tomato plants with *GMEs* (*SlGME1* and *SlGME2*) overexpression or suppression exhibit increased or decreased AsA concentration, respectively [24,25]. The effects of inhibiting *SlGME1* and *SlGME2* on ascorbate content showed a consistent function, but only *SlGME1* but not *SlGME2* affect the pollen development and pollination process [26]. The enzymatic reaction of GGP is the first step in the particular trail of d-Man/l-Gal pathway different from the d-gluconose pathway and is involved in the conversion of GDP-l-galactose into l-galactose-1-phosphate. Three *GGP* defective mutants, *vtc2-1*, *vtc2-2*, and *vtc2-3* were screened in ethyl methanesulfonate (EMS)-mutagenized *Arabidopsis* seedlings by nitroblue tetrazolium (NBT)-based assay [16]. Additionally, *vtc5* mutant (a close homolog of *VTC2*) was isolated and resulted in a decreased accumulation (80% of the wild type) of AsA *Arabidopsis* [17]. Moreover, the double mutant (*vtc2*/*vtc5*) showed even less ascorbate accumulation, resulting in immediate growth arrest upon germination and cotyledon bleaching, while normal growth was restored by supplementation with ascorbate or l-Gal [17]. *GPP,* named as *VTC4* in *Arabidopsis*, converts l-galactose-1-phosphate to l-galactose. The *vtc4* mutant showed less myo-inositol and AsA accumulated, indicating that VTC4 is a bifunctional enzyme that impacts both myo-inositol and AsA synthesis pathways [27]. The expression analysis of AsA related genes suggested that *GPP* plays an important role in the regulation of AsA accumulation in tomato [28]. GalDH has a very high conversion efficiency for l-Gal, and can quickly convert exogenous l-Gal into ascorbate [10]. Therefore, the GalDH catalyzed reaction is not a rate-limiting step in the ascorbate synthesis pathway. Knock-down of the expression of *GLDH* doesn’t affect the content of ascorbate in both leaves and fruits of tomato suggesting GLDH is also not a rate-limiting enzyme for ascorbate synthesis [1].

Besides structural genes, many factors such as light, temperature, ozone, hormones, and regulatory factors can regulate AsA accumulation [29]. For instance, high light, low temperature, and combined high light and low-temperature treatment can increase AsA content by 10–50% in *Dunaliella* species [30,31]. In tomato, seven days of shading resulted in a 50% reduction of AsA in leaves and a 10% decrease in antioxidant activity in fruit [32]. AMR1 (ascorbic acid mannose pathway regulator 1) is a regulatory factor with an F-box structure that affects the expression of genes in d-Man/l-Gal pathway and showed a negative regulation of AsA levels and ozone-nonsensitivity in *Arabidopsis* [33]. AtERF98 and SlHZ24, as transcription factors, directly bind to the promoter of *VTC1* and *SlGMP3* and affect the AsA accumulation in Arabidopsis and tomato, respectively [34,35]. 

It is well-known that many of the plant traits are controlled by multigenes, especially for metabolites due to the complex metabolic processes of biosynthesis, degradation, and recycling [36,37,38]. For complex biosynthetic pathways or multiple traits; in some cases, engineering a single gene is insufficient to improve the yield of metabolic products; the assembly of multigene expression is often required. To achieve this purpose, researchers have proposed several pyramiding methods, such as cotransformation of multiple genes and transgenic pyramiding by conventional hybridization. Several examples have been developed via cotransformation with a few regulatory or structural genes of the target metabolic biosynthesis pathways, such as β-carotene-enriched “Golden Rice” [39] and anthocyanin-enriched “Purple Tomatoes” and “Purple Endosperm Rice” [36,40]. Pyramiding by conventional hybridization is time-consuming compared with cotransformation. However, pyramiding by hybridization is technologically feasible and generate stable inherited target genes [41]. It has been also reported that transgenic pyramiding improves plant salt tolerance, disease resistance, and amino acid content [42,43,44]. 

Unlike the anthocyanin or carotenoids pathways, there is no research on the application of biosynthetic pyramiding in AsA pathway. In this study, to further investigate the AsA biosynthesis and its engineering in tomato, we pyramided structural genes (SGs) involved in d-Man/l-Gal AsA pathway by conventional hybridization. Functional characterization of different pyramiding transgenic lines showed significantly enhanced AsA levels, light sensitivity, AsA transport capacity, and tolerance to oxidative stresses.

## 2. Results

### 2.1. Gene Pyramiding Altered Expression of AsA-Related Genes and AsA Concentration in Both Leaves and Fruits

**Figure 1:**
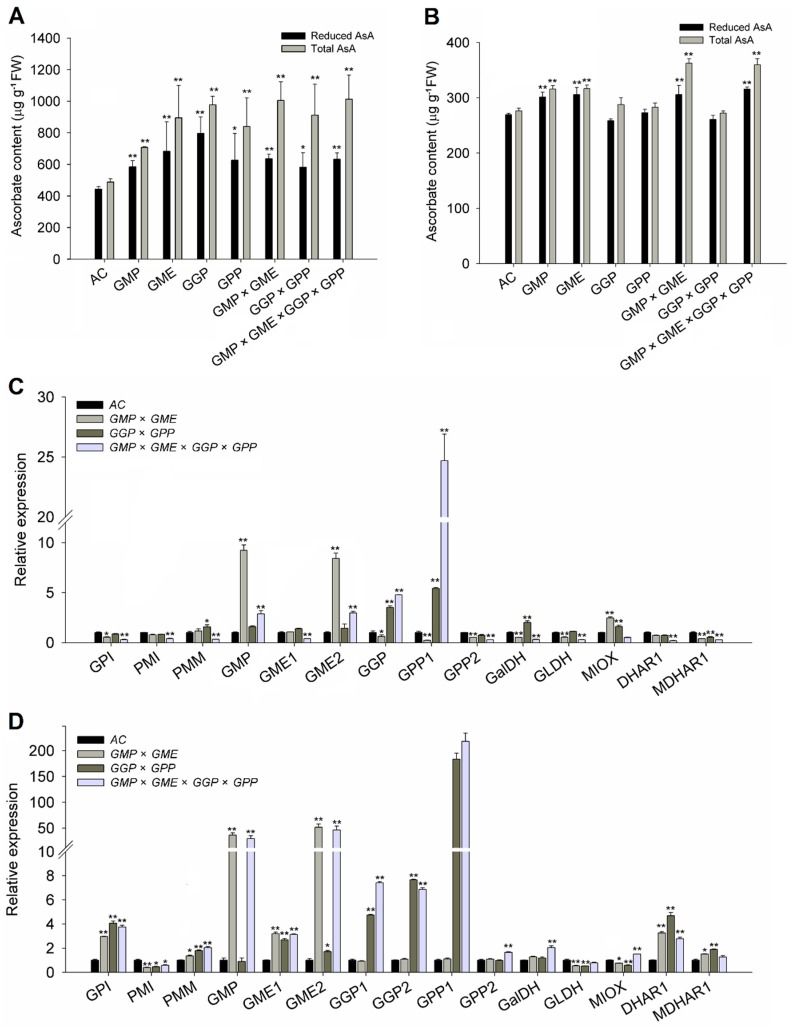
Relative expression of AsA biosynthesis, recycling-related genes, and ascorbate concentration in leaf and red ripe fruit of transgenic and pyramiding tomato lines. (**A**) Reduced and total ascorbate content in young leaf. (**B**) Reduced and total ascorbate contents in red ripe fruit. (**C**) Relative expression of AsA biosynthesis and recycling-related genes in leaf. (**D**) Relative expression of AsA biosynthesis and recycling-related genes of red ripe fruit. *GPI* (glucose-phosphate isomerase, Solyc04g076090), *PMI* (phosphomannose isomerase, Solyc02g086090), *PMM* (phosphomannomutase, Solyc05g048760), *GMP* (GDP-Man pyrophosphorylase, Solyc03g096730), *GME1* (GDP-Man-3′,5′-epimerase 1, Solyc01g097340), *GME2* (GDP-Man-3′,5′-epimerase 2, Solyc04g077020), *GGP1* (GDP-l-Gal phosphorylase/l-Gal guanylyltransferase 1, Solyc06g073320), *GGP2* (GDP-l-Gal phosphorylase/l-Gal guanylyltransferase 2, Solyc02g091510), *GPP1* (l-Gal 1-phosphate phosphatase 1, Solyc04g014800), *GPP2* (l-Gal 1-phosphate phosphatase 2, Solyc11g012410), *GalDH* (l-Gal dehydrogenase, Solyc01g106450), *GLDH* (l-GalL dehydrogenase, Solyc10g079470), *MIOX* (*myo*-inositol oxygenase, Solyc12g008650), *MDHAR1* (monodehydroascorbate reductase, Solyc09g009390), *DHAR1* (dehydroascorbate reductase, Solyc05g054760). Three replicate experiments were performed. Error bars represent standard error, means ± SE. FW, fresh weight. The asterisks represented significant differences from wild type (AC), as indicated by the *t*-test (* *p* < 0.05; ** *p* < 0.01).

The *GMP × GME* and *GGP × GPP* pyramiding lines were generated by conventional crossing of *GMP* with *GME* and *GGP* with *GPP*, respectively. Then, in the same way, four gene pyramiding lines (*GMP × GME × GGP × GPP*) were generated. The expression of AsA-related genes and content of AsA were measured in the wild-type and three pyramiding lines (Figure 1). In leaves, the total AsA and reduced AsA levels in *GGP* overexpression line and *GMP × GME*, *GMP × GME × GGP × GPP* pyramiding lines showed a 2-fold increase compared with wild-type, and significant increase was also observed in *GMP*, *GME*, *GPP*, *GGP × GPP* lines (Figure 1A). The considerable amount of ascorbate in *GME*, *GGP*, *GPP*, *GMP × GME*, *GGP × GPP,* and *GMP × GME × GGP × GPP* indicated that there is a limit in the increase in AsA content in tomato leaves and that the biosynthesis of AsA may be subjected to feedback inhibition. The ascorbate content in fruits in *GME*, *GMP* overexpression lines, and *GMP × GME*, *GMP × GME × GGP × GPP* pyramiding lines increased significantly compared with the wild-type, while no significant ascorbate content changes were observed in *GGP*, *GPP* and *GGP×GPP* lines (Figure 1B). 

In addition to the increased expression of the pyramided genes themselves in the three pyramiding lines, such as the increased expression of *GME2* and *GMP* in *GMP × GME* pyramiding line and increased expression of *GGP* and *GPP1* in *GGP × GPP* pyramiding lines, the transcript level of other AsA biosynthetic and recycling genes showed slight or significant decline, especially in leaves of the *GMP × GME × GGP × GPP* pyramiding line (Figure 1C). In fruits, however, except *PMI*, *GLDH*, and *MIOX*, the expression of most AsA-related structural genes, such as the *GPI*, *GMP*, *GME1*, *GME2*, *GPP1*, and *DHAR1*, showed a significant increase in three pyramiding lines (Figure 1D). Taken together, we speculated that in the d-Man/l-Gal pathway, all *GMP*, *GME*, *GGP*, and *GPP* contribute to the biosynthesis of AsA in leaves, and *GGP* and *GPP* function in AsA accumulation in tomato leaf more efficiently than in fruits. The biosynthesis of AsA may be subjected to feedback inhibition, that is, overexpression of the target gene in the pyramiding lines increases the content of AsA, but downregulates the expression of other structural genes by feedback inhibition. 

### 2.2. Different Biosynthesis Pathways Contributed to the Total AsA Content in Leaves and Fruit of Pyramiding Lines

According to the above-mentioned results, we assumed that increase in the AsA content after pyramiding is connected with the contribution of different AsA biosynthesis pathways in leaves and fruits. Thus, three substances representing the three biosynthesis pathways, inositol (MI) corresponding to inositol pathway, glucose (GLc) corresponding to d-Man/l-Gal pathway and galacturonic acid (GLA) corresponding to the galacturonic acid pathway, were selected for feeding experiments. AsA and H_2_O were utilized as a positive and negative controls, respectively. In leaves, feeding with MI and GLA significantly decreased AsA content in both pyramiding lines and AC. On the contrary, the AsA content in wild-type and pyramiding lines were significantly increased by feeding with GLc which is the primary product of photosynthesis in leaves and a precursor of d-Man/l-Gal pathway (Table 1). Glucose plays a decisive role in AsA biosynthesis suggesting that d-Man/l-Gal pathway was the main contributor of AsA accumulation in tomato leaves. 

Feeding with both MI and GLc can increase the content of AsA in fruits of pyramiding lines. However, the AsA content in the fruit of AC and the *GGP × GPP* pyramiding line showed no change after feeding with GLA (Table 1). This result suggested that d-Man/l-Gal pathway, the d-galacturonate pathway, and the myo-inositol pathway all contributed to AsA biosynthesis in tomato red fruit in pyramiding lines. Taken together, gene pyramiding enhanced the AsA biosynthesis pathway in which the d-Man/l-Gal pathway plays a role in leaves and the three pathways all function in fruits, and subsequently affect AsA content. 

### 2.3. Light-Dependent Fluctuation of AsA Content in Wild Type and Pyramiding Lines

It has been reported that light intensity is one of the critical environmental factors affecting AsA accumulation [29], and the feeding experiment with its precursors in leaves and fruits indicated that glucose, as a product of photosynthesis, had a significant influence on the AsA content in leaves. To further study the effect of pyramiding on light-dependent ascorbate metabolism, we measured the AsA content in leaves under different light treatments for 48 h.

**Figure 2:**
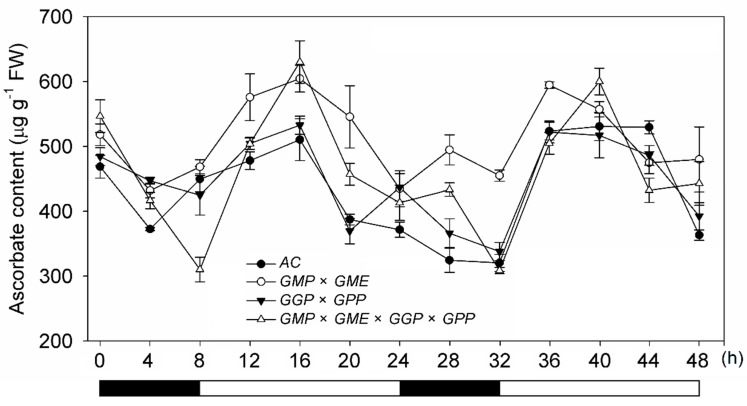
Dynamic change of ascorbate accumulation in response to light in leaves of AC and pyramiding lines. Plants were grown in the greenhouse, and fully expanded leaves were harvested every 4 h, total ascorbate content was measured for 48 h (8 dark/16 light). Three replicate experiments were performed. Error bars represent standard error, means ± SE. FW—fresh weight.

The total AsA content exhibited similar dynamic changes, as a model of “down-up-down-up-down”, in both wild-type and pyramiding lines under different light treatments for 48 h. Therefore, when we compared the AsA content in AC and *GGP × GPP* line, the AsA content in *GMP × GME*, and *GMP × GME × GGP × GPP* lines were higher at most of the time points and had larger fluctuations (Figure 2). These findings suggested that the accumulation of AsA in tomato showed a specific response to photoperiod, and light is indeed an important environmental factor affecting AsA levels. The AsA content in leaves increased at the first stage of light treatment but decreased during further illumination, suggesting that the accumulation of AsA maximizes within a certain light frame (8 h, 6:00 to 14:00), but will not further increase with the extension of light. Taken together, gene pyramiding enhanced the light-induced AsA accumulation in tomato leaves and the fluctuation amplitude. 

### 2.4. Pyramiding Biosynthetic Genes Increases AsA Transport Capacity

**Figure 3:**
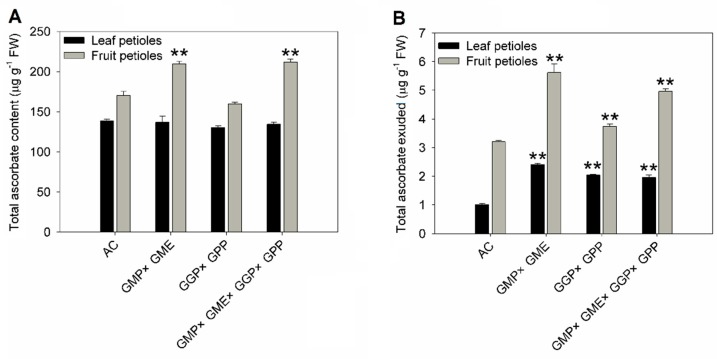
Ascorbate concentration in leaf and fruit petioles and their exudates of wild-type and pyramiding tomato lines. (**A**) AsA levels in fruit petioles and leaf petioles of wild type and pyramiding lines. (**B**) AsA content in exudates of fruit and leaf petioles in AC and pyramiding lines. Three replicate experiments were performed. Error bars represent standard error, means ± SE. FW—fresh weight. The asterisks represented significant differences from wild-type (AC), as indicated by the *t*-test (** *p* < 0.01).

It has been reported that AsA can be synthesized in both leaves and fruits and transported from leaves to fruits [45]. In order to investigate how pyramiding affects AsA transport capacity, we designed two experiments. First, AsA content was measured in leaf and fruit petioles of wild-type and pyramiding lines. The content of AsA showed no significant difference in leaf petioles between wild-type and pyramiding lines, but showed a significant increase in fruit petioles of *GMP × GME* and *GMP × GME × GGP × GPP* lines, respectively (Figure 3A), consistent with the AsA content in the red fruit (Figure 1B). The AsA content in the exudates of fruit and leaf petioles were also measured in AC and pyramiding lines. The results showed that the AsA content significantly increased in the secretion of both fruit and leaf petioles in pyramiding lines compared with AC (Figure 3B).

**Figure 4:**
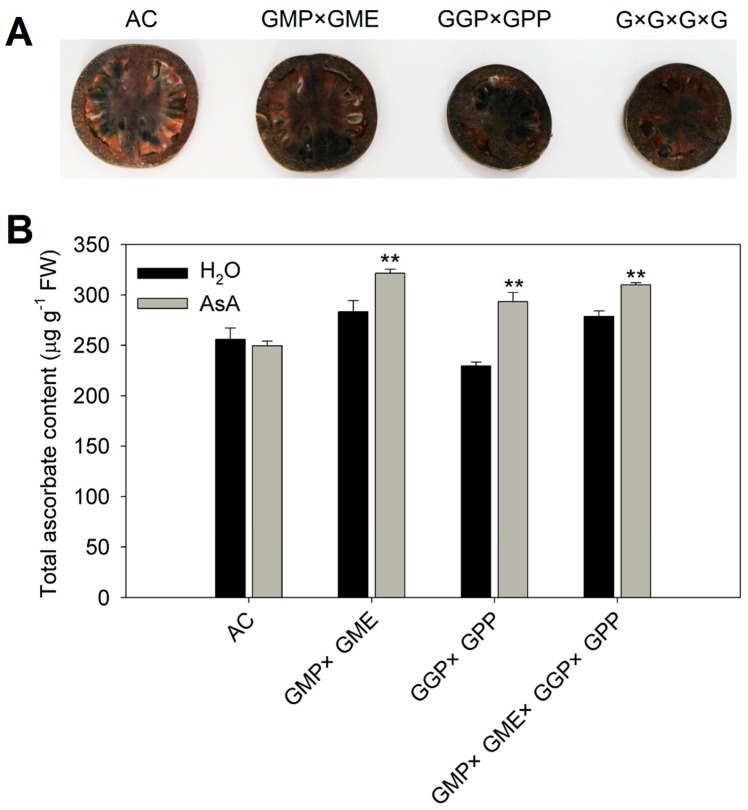
Ascorbate content in fruits of AC and pyramiding lines after fruit petioles cultured in 5 mM AsA for 24 h. (**A**) AsA localization using AgNO_3_ in transverse sections of mature green fruit after fruit petioles were cultured in 5 mM AsA for 24 h. (**B**) AsA levels in the fruit of AC and pyramiding lines after fruit petioles were cultured in 5 mM AsA for 24 h. Three replicate experiments were performed. Error bars represent standard error, means ± SE. FW—fresh weight. The asterisks represented significant differences from control (H_2_O), as indicated by the *t*-test (** *p* < 0.01).

Next, the petioles of mature green fruits were incubated with 5 mM AsA (water as a control) for 24 h at 16 light/8 dark, and the AsA content of fruit was measured. AsA concentration showed no change after feeding with AsA compared with water in the fruit of AC, but the content of AsA showed a significant increase in *GMP × GME*, *GGP × GPP* and *GMP × GME × GGP × GPP* pyramiding lines, suggesting that pyramiding lines possessed stronger AsA transport capacity than AC (Figure 4B). To further detect the AsA content and its distribution in the fruits for which the petioles were incubated with 5 mM AsA, we investigated the localization of reduced AsA by AgNO_3_ histochemical localization where the reduced AsA can react with AgNO_3_ to form metallic Ag, and the content and distribution of “black” Ag ions represents the AsA. In AC fruit, Ag accumulation mainly locates in the inner wall of the pericarp, placental tissue, and seeds; but the distribution of Ag is broader, and its concentration is higher in the fruits of *GMP × GME, GGP × GPP,* and *GMP × GME × GGP × GPP* pyramiding lines (Figure 4A), consistent with the results shown in Figure 4B. All of these results indicated that gene pyramiding increases AsA transport capacity in both leaves and fruit of tomato.

### 2.5. AsA Biosynthetic Pyramiding Improved Tolerance to Oxidative Stress in Tomato

**Figure 5:**
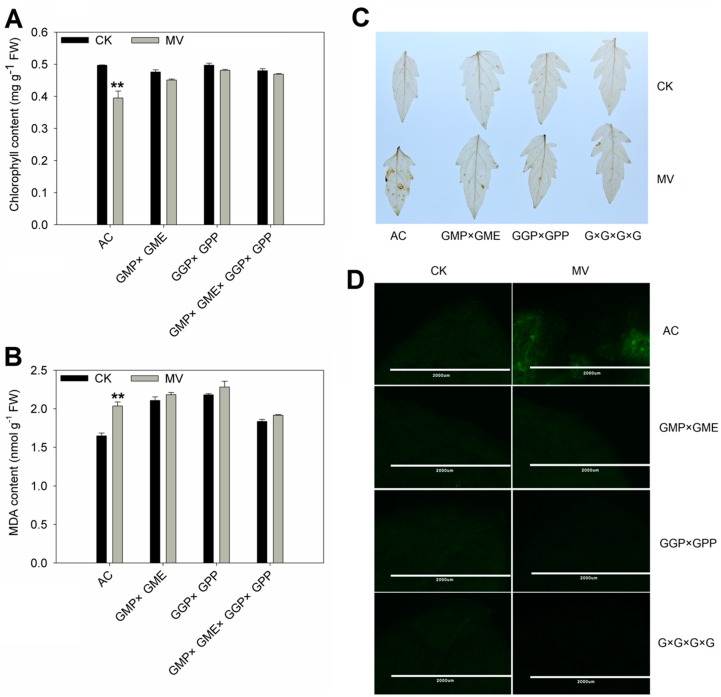
The oxidative stress tolerance in AC and pyramiding tomato lines. (**A**,**B**) The chlorophyll (**A**) and malondialdehyde (MDA) content (**B**) in leaves treated with methyl viologen (MV) or water (CK) was measured on days 3 and 7 post-treatment, respectively. Three replicate experiments were performed. The data presented are means ± SE. The asterisks represent significant differences from the control (CK), as indicated by the *t*-test (** *p* < 0.01). (**C**) Detection of H_2_O_2_ accumulation in the leaves of AC and pyramiding lines by DAB staining in leaves from the two-month-old plants of AC and pyramiding lines. (**D**) H_2_DCFDA fluorescence (bar = 2000 μm) to reveal the accumulation of H_2_O_2_ in leaves from the two-month-old plants of AC and pyramiding lines. *G × G × G × G* means *GMP × GME × GGP × GPP*.

AsA is an antioxidant molecule that removes the ROS and protects plants from oxidative damage. In most cases, increased AsA content can improve plant resistance to oxidative stress. To evaluate whether pyramiding of AsA-related structural genes in tomato can increase tolerance to oxidative stress, 1-month-old pyramiding lines and AC were subjected to oxidative stress by spraying with 75 μM methyl viologen (MV), which can simulate the oxidation environment, for two days. The degree of leaf chlorophyll loss reflects the damage caused by MV. As shown in Figure 5, chlorophyll content showed no significant difference between AC and pyramiding lines without MV treatment. After MV treatment, chlorophyll content in the AC plants showed a significant decrease (20.5%), whereas no significant decrease was found in pyramiding lines (5.3% in *GMP × GME*, 3.3% in *GGP × GPP*, and 2.3% in *GMP × GME × GGP × GPP*) (Figure 5A). After MV treatment, MDA content was significantly increased in AC, while no significant change in MDA was observed in pyramiding lines (Figure 5B). Moreover, DAB staining and H_2_DCFDA fluorescence observation were further conducted to detect the accumulation of H_2_O_2_ in leaves of AC and pyramiding lines. DAB staining showed that there was no significant difference between AC and pyramiding plants after treatment with H_2_O, but more brown spots in the leaves of AC were observed than in the leaves of pyramiding lines after MV treatment (Figure 5C). A similar result was observed by H_2_DCFDA fluorescence; no significant difference was found between AC and pyramiding tomato lines treated with H_2_O, but AC generated brighter fluorescence than pyramiding lines after being treated with MV (Figure 5D). Taken together, all these results demonstrate that transgenic pyramiding of AsA-related structural genes in tomato increased tolerance to oxidative stress. In addition, under a natural chilling injury, pyramiding lines showed stronger cold resistance and plants recovered to normal growth more quickly and generated new leaves earlier.

### 2.6. The Fruit Shape, Fruit Size, and Soluble Solids Were Affected by Gene Pyramiding

**Figure 6:**
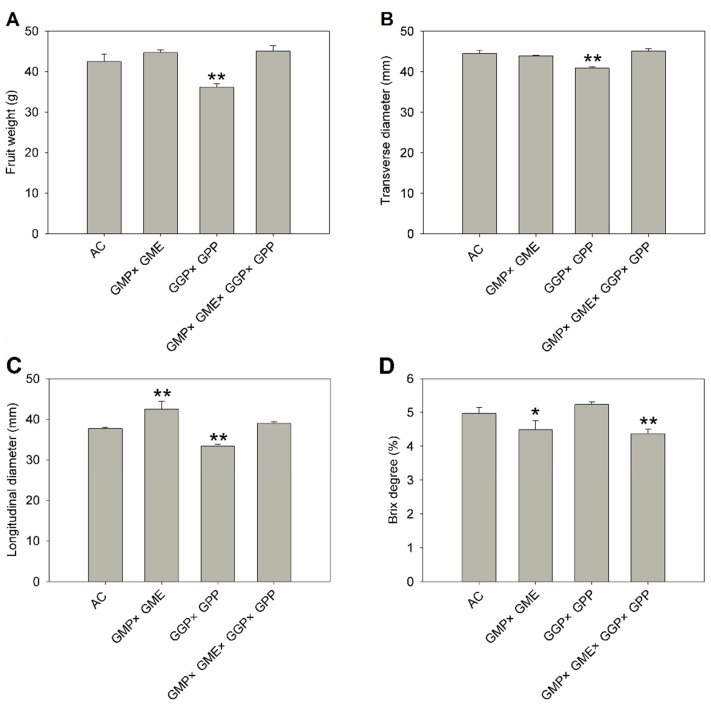
The fruit weight (**A**), transverse diameter (**B**), longitudinal diameter (**C**), and brix degree (**D**) in AC and pyramiding lines. Three replicate experiments were performed. Error bars represent standard error, means ± SE. *G × G × G × G* means *GMP × GME × GGP × GPP*. The asterisks represented significant differences from wild type (AC), as indicated by the *t*-test (* *p* < 0.05; ** *p* < 0.01).

In addition to enhanced AsA accumulation, the three pyramiding lines showed obvious differences in fruit shape, size, and brix degree compared with AC (Figure 6). The fruits of the *GMP × GME* line showed increased vertical diameter but no difference in longitudinal diameter and fruit weight, leading to oval round fruit. The fruit weight, fruit diameter, longitudinal diameter of *GGP × GPP* lines was significantly reduced compared to AC (Figure 6). The brix degree of *GMP × GME* and *GMP × GME × GGP × GPP* lines were significantly lower than AC (Figure 6). These findings showed that pyramiding of AsA related structural genes could affect the appearance and brix of tomato fruit. 

## 3. Discussion

In the past decades, many studies on genetic engineering of plant disease resistance, drought resistance, and salt tolerance have been reported [42,44,46,47]. Also, using genetic engineering methods to develop food crops with increased contents of specific micronutrients and phytonutrients such as high-zinc rice, provitamin A-enriched maize, and anthocyanin-enriched “Purple Tomatoes’’, have been well developed [36,41]. However, the genetic engineering of AsA pathway has rarely been reported. In this study, effective biofortification for improving AsA content in tomato has been conducted by pyramiding *GMP × GME*, *GGP × GPP*, and tetravalent pyramiding *GMP × GME × GGP × GPP*. 

*GMP* [22], *GME* [26], *GGP* [48], and *GPP* [49] are the major genes in the d-Man/l-Gal pathway of ascorbate biosynthesis and engineering of these genes can improve the AsA content to an extended degree. Our results showed that the increase in AsA content of pyramiding lines was comparable to the single-gene overexpression lines, e.g., *GMP* overexpressing lines (Figure 1), suggesting that AsA accumulation will not increase without limit by gene pyramiding in tomato. We hypothesize a rate-limiting step for AsA synthesis and self-regulating mechanism for AsA homeostasis. 

In this study, a series of experiments were conducted to reveal the molecular mechanism for the increase in AsA content in plants of pyramiding AsA-related genes. The possible reasons for the increase of AsA content in pyramiding lines were assumed to be multifactorial. First, compared with AC, the transcript level of most AsA biosynthesis-related genes were upregulated in the leaves and red ripened fruits of pyramiding lines (Figure 1A,C). Therefore, the activated AsA pathway may be one of the reasons for the increase of AsA content in pyramiding lines. Second, pyramiding did not completely break the basic rule of the circadian rhythm of AsA but increased the amplitude of fluctuation during the 48 h period (Figure 2). We assumed that the pyramiding enhances plant sensitivity to light and increases the product of photosynthesis substrate, which is involved in the main biosynthetic pathway of AsA [50], and thus increases the accumulation of AsA. Third, a precursor feeding experiment showed that the d-Man/l-Gal pathway especially contributes to AsA synthesis in the leaves of AC and pyramiding tomato lines. However, besides contribution of d-Man/l-Gal pathway and myo-inositol pathway to AsA biosynthesis in both AC and pyramiding lines, d-galacturonate pathway also plays a vital role in the synthesis of AsA in the fruits of the *GMP × GME* and *GMP × GME × GGP × GPP* pyramiding plants (Table 1 and Figure 4B). This is the reason why AC and *GGP × GPP* plants showed lower AsA content than *GMP × GME* and *GMP × GME × GGP × GPP* plants (Figure 1B). Besides, pyramiding biosynthetic genes also increased the transport capacity of AsA. Taken together, key structural gene pyramiding enhances synthesis and transport capacity of AsA in tomato. 

Ascorbate is a potent oxidizing agent and the increase of ascorbate can enhance plant resistance to biotic and abiotic stress [51]. In this study, we evaluated the effect of gene pyramiding on abiotic stress. The results showed that with the increase of AsA, the tolerance to oxidative stress was enhanced in pyramiding lines, consistent with previous results [3,51]. In addition to enhanced antioxidant capacity, pyramiding also affects some of the agricultural traits, such as the fruit shape, fruit size, and soluble solids (Figure 6). Photosynthesis products are utilized as sources for ascorbate biosynthesis, and link ascorbate to sugar and organic acid metabolism in tomato [52]. Genetic engineering of the ascorbate pathway may affect other primary metabolism pathways. Previous studies have shown that *GME* plays a crucial role in both cell wall and ascorbate metabolism. *GME*-RNAi plants showed reduced fruit size due to the less cell size than the control [24]. In this study, several primary metabolism pathways, including sugar, acid, and cell wall metabolism, may contribute to different fruit shape, fruit size, and soluble solids in pyramiding lines. Further investigation is required to define this AsA regulation by pyramiding genes in tomato regarding how it may contribute to the taste (soluble solids) and yield (fruit shape and size) of the fruits. 

## 4. Methods

### 4.1. Plant Growth Conditions and Sampling

The vectors containing the complete ORFs of *GMP, GME, GGP*, or *GPP* were introduced into pMV expression vector under the CaMV35S promoter, and confirmed by sequencing and transformed into *Agrobacterium tumefaciens* C58 by electroporation. Tomato seeds of wild type AC (Ailsa Craig) and the *GMP* (Solyc03g096730)*, GME* (Solyc04g077020)*, GGP* (Solyc06g073320)*, GPP* (Solyc04g014800) over-expression lines were preserved in our laboratory. Tomato plants were germinated and planted in a naturally illuminated glasshouse. Tissues from leaves (fifth leaf from top), pericarp of red fruits, petioles of leaf, and fruit of control and pyramiding plants were collected. Three biological replicates of each line were analyzed, and each biological replicate contained three individual samples (leaves, fruits, and petioles of leaf and fruit) of the same developmental stage from the same genotype. Samples were immediately frozen in liquid nitrogen and stored at −80 °C for further AsA assay and RNA isolation.

### 4.2. RNA Isolation and Gene Expression Analysis

Total RNA was extracted from various tissues of pyramiding lines and wild-type plants using TRIZOL reagent (Invitrogen, Waltham, MA, USA). Approximately 100 mg powdered leaves and 200 mg powdered fruits were used for RNA extraction. Complementary DNAs were synthesized from total RNA using the HiScript® II Reverse Transcriptase (Vazyme, Miramar Beach, FL, USA) following the manufacturer’s protocol. Quantitative real-time PCR (qRT-PCR) analyses were performed with LightCycler® 96 SW 1.1 using the LightCycler480 SYBR Green I Master Kit (Roche, Basel, Switzerland, http://www.roche.com/) according to the manufacturer’s protocols. Primer sequences for AsA metabolism-related genes are listed in Appendix A2. Three biological replicates for pyramiding lines and the wild type were performed. The relative expression of specific genes was measured using the cycle threshold 2^-ΔΔ*C*t^ method. The actin gene (BT013524) was used as a constitutive internal control.

### 4.3. Determination of AsA Content

Approximately 200 mg of powdered leaf sample and 400 mg of powdered fruit sample were used for total AsA and reduced AsA determination, as previously described [35]. Briefly, samples were collected and grounded in liquid nitrogen and homogenized in 1 mL of ice-cold 6% trichloroacetic acid (TCA). After 15 min extraction on ice, for total AsA levels, 20 μL of the supernatant was transferred to wells of a microtiter plate containing 20 μL of 5 mM dithiothreitol (DTT). After 20 min incubation at 37 °C, 10 μL of *N-*ethylmaleimide (NEM) was added, and followed by incubation for 1 min at room temperature. 8 μL of the color reagent was then added to the mixture and incubate for 1 h at 37 °C. Ascorbate was detected at 550 nm using an InfiniteM200 (Tecan, Männedorf, Switzerland, http://www.tecan.com/). For reduced AsA, replaced the DTT and NEM by the same volume of 0.4 mol potassium phosphate buffer (pH 7.4) while the rest of the procedure was as for the total AsA assay. All used reagents were prepared as previously described [35].

### 4.4. Synthetic Precursor Feeding to Leaves and Fruits

The one-month-old wild type (Ailsa Craig, AC) and pyramiding lines were used for synthetic precursor feeding of leaves by the leaf-disc method. The cut leaves by puncher were placed in a Petri dish, containing 20 mL of 5 mM MI (Inositol), GLc (glucose), GLA (Galacturonic acid), and AsA, respectively. Leaves incubated in water as a control. The petri dishes were placed in a growth chamber under a 16 h/8 h light/dark cycle at 25 °C for 24 h. After incubation, leaves were washed three times with distilled water, gently dried, and then frozen with liquid nitrogen and stored at −80 °C. For each line, three biological replicates were analyzed. For red fruit feeding analysis, the fruit stalk instead of leaf-disc was dipped in 20 mL of 5 mM MI (Inositol), GLc (glucose), GLA (Galacturonic acid), and AsA, respectively. Fruits incubated in water were used as a control. At the end of the incubation period, the stalk was detached from the fruit, and the fruit was washed with distilled water twice and mopped gently, and the pericarp was frozen with liquid nitrogen and stored at −80 °C for further analysis.

### 4.5. Light Response Assay

The one-month-old wild type (AC) and pyramiding lines were grown in a greenhouse under a 16 h/8 h light/dark cycle at 25 °C. For the light response characterization, the plants were exposed to continuous light at 25 °C for 16 h followed by 8 h continuous dark under 25 °C. In a 48-h photoperiod, samples were taken at 0, 4, 8, 12, 16, 20, 24, 28, 32, 36, 40, 44, and 48 h (0, 4, 8, 24, 28, and 32 h under dark, 12, 16, 20, 36, 40, 44, and 48 h under light) to determine the content of total AsA in tomato leaves. 

### 4.6. Oxidative Stress Treatment

To evaluate the performance of pyramiding lines under oxidative stress, leaves of AC and pyramiding lines were thoroughly sprayed with 75 μM methyl violet (MV) (MV dissolved in water with 0.1% Tween-20) or water with 0.1% Tween-20 (control) once a day for 2 days. After treatment for one week, leaves were collected and frozen with liquid nitrogen for the determination of chlorophyll, malondialdehyde (MDA). Also, the treated fresh leaves are used for DAB staining and H_2_DCFDA fluorescence.

For the measurement of chlorophyll content, 0.2 g frozen leaf was extracted with 1.5 mL of 80% (*v/v*) acetone. Extraction was performed under low light intensity. Absorption of the extracts at 663 (Chl*a*) and 646 (Chl*b*) nm were measured using DU800 UV/Vis Spectrophotometers (Beckman Coulter, Brea, California, USA). Chlorophyll was quantified using the following equation: Chlorophyll concentration (mg/mL) = 6.63 A_665_ + 18.08 A_649_, where “A” represents absorbance at the specified wavelength. 

For the measurement of MDA content, 0.2 g frozen leaf was extracted with 3 mL of 5% trichloroacetic acid (TCA). Absorption of the extracts at 50 nm, 532 nm, and 600 nm were measured using DU800 UV/Vis Spectrophotometers (Beckman Coulter). MDA was quantified using the following equation: MDA concentration (μM/L) = 6.45 (A_532_-A_600_) -0.56 A_450_, where “A” represents absorbance at the specified wavelength. 

MV treated leaf samples were stained with 3, 3′-diaminobenzidine (DAB) solution to detect the presence of H_2_O_2_, as previously described [53].

For H_2_DCFDA fluorescence analysis, MV treated leaf samples were immersed in 25 μM H_2_DCFDA solution for 15 min in dark place, and then washed three times with 20 mM phosphate buffer (pH 6). Finally, H_2_DCFDA fluorescence was detected and photographed using a non-eyepiece fluorescence microscope (NIKON ECLIPSE 80i, Tokyo, Japan). 

### 4.7. The AsA of Exudates of Leaf and Fruit Petioles Phloem

The leaf and fruit petioles were taken from pyramiding lines and AC plants at the same developmental stage and the same position, then washed with distilled water twice and mopped gently, and cut into the 1 cm segment in water, then surface dried on absorbent cotton. The cut end was put into the 1 mL of 15 mM EDTA (pH 7.5) in 5 mL centrifuge tube, incubation for 5 h at 25 °C in relative humidity 90%, and then the supernatant was used to measure AsA content as described above. 

### 4.8. Histochemical Staining of AsA with Acidic AgNO_3_

The acidic–alcoholic AgNO_3_ method was used for histochemical localization of AsA, as previously described [54]. Briefly, the mature green fruits were longitudinal cut about 2 mm thick and stained by 5% of ice AgNO_3_ solution containing a mixture of ethanol: acetic acid: H_2_O (66:10:29, *v/v/v*)) with for 24 h at 4 °C. Then the treated tissues were rinsed with 70% ethanol solution containing 5% of ammonia for at least three times. Finally, the sample was continuously immersed in the cleaning solution and stored in 70% ethanol solution at 4 °C. The images were captured with an ordinary Nikon SLR camera.

### 4.9. Data Analysis

For all experiments, samples were biologically replicated at least three times, and results were represented as means with standard error. If two observations were described in the text as statistically significant, it was calculated by Student’s *t*-test at the *p* < 0.05 and *p* < 0.01 levels using SPSS software (http://www-01.ibm.com/software/analytics/spss/). Calculations were carried out with Microsoft Excel software and Graphics were implemented with Excel and SigmaPlot (San Jose, CA, USA, SYSTAT.SigmaPlot.v10.0).

## 5. Conclusions

Although the functions of critical structural genes involved in the ascorbate biosynthetic pathway have already been characterized in several plant species, structural gene pyramiding in horticultural crops, especially in those with fleshy fruits such as tomato, remains to be further studied. In this study, four key genes, *GME*, *GMP*, *GGP*, and *GPP*, were pyramided in tomato by conventional hybridization. Pyramiding lines exhibited increased AsA content, along with enhanced light response, stress tolerance, and AsA transport capacity. Additionally, fruit shape, fruit size, and soluble solids were slightly affected by pyramiding. Substrate feeding reveals different ascorbate biosynthesis pathways in tomato leaves and fruit. The data shown here should be taken into consideration in future studies for genetic improvements of fruit quality and/or stress tolerance in tomato.

## Figures and Tables

**Table 1:** Effect of feeding AsA biosynthesis precursors on the content of ascorbate in tomato.

**Lines**	Feeding-leaf- total ascorbate content (μg g^−1^ FW)				
	**H_2_O**	**MI**	**GLc**	**GLA**	**AsA**
AC	496.2 ± 6.3	422.5 ± 15.9 **	591.2 ± 7.6 **	215.6 ± 8.5 **	2139.9 ± 59.9 **
*GMP* × *GME*	554.6 ± 14.2	452.4 ± 14.2 **	742.4 ± 10.5 **	162.5 ± 9.9 **	2733.7 ± 291.6 **
*GGP* × *GPP*	579.8 ± 15.9	427.8 ± 3.2 **	623.8 ± 6.5 **	158.0 ± 9.8 **	2220.3 ± 71.9 **
*GMP* × *GME* × *GGP* × *GPP*	626.1 ± 10.2	508.1 ± 17.8 **	790.9 ± 6.4 **	283.1 ± 13.3 **	2584.4 ± 72.2 **
**Lines**	Feeding-fruit- total ascorbate content (μg g^−1^ FW)				
	**H_2_O**	**MI**	**GLc**	**GLA**	**AsA**
AC	254.3 ± 3.6	276.1 ± 1.4 *	287.9 ± 3.0 **	259.9 ± 5.2	296.3 ± 1.2 **
*GMP* × *GME*	287.5 ± 4.0	384.6 ± 8.0 *	396.2 ± 2.5 **	325.8 ± 4.9 **	368.4 ± 11.3 **
*GGP* × *GPP*	251.0 ± 2.8	265.9 ± 4.7 *	350.1 ± 6.9 **	261.9 ± 6.1	351.5 ± 34.8 **
*GMP* × *GME* × *GGP* × *GPP*	301.9 ± 8.3	373.7 ± 5.9 **	390.4 ± 7.4 **	331.5 ± 5.4 **	354.0 ± 7.0 **

The leaves (upper panel) and fruits (lower panel) were fed with H_2_O, inositol (MI), glucose (GLc), galacturonic acid (GLA), and ascorbate (AsA) and incubated under light for 24 h. Three replicate experiments were performed. The data presented are means ± SE. FW—fresh weight. The asterisks represented significant differences from wild type (AC), as indicated by the *t*-test (* *p* < 0.05; ** *p* < 0.01).

## Appendix A

A1: Proposed ascorbate biosynthetic pathways in plants.

The four boxes show *myo*-inositol pathway, d-gulose pathway, d-mannose/l-galactose pathway and d-galacturonate pathway from left to right. Enzymes: 1, GDP-Man pyrophosphorylase; 2, GDP-Man-3′,5′-epimerase; 3, GDP-l-Gal phosphorylase/l-Gal guanylyltransferase; 4, l-Gal 1-phosphate phosphatase; 5, l-Gal dehydrogenase; 6, l-GalL dehydrogenase; 7, UDP-glucuronate 4-epimerase; 8, UDP-galacturonate pyrophosphatase or phosphorylase or uridylyltransferase; 9, d-galacturonate 1-phosphate phosphatase; 10, d-galacturonate reductase; 11, aldonolactonase; 12, *myo*-inositol oxygenase; 13, d-glucuronate reductase; 14, l-GulL dehydrogenase or oxidase; 15, ascorbate peroxidase; 16, monodehydroascorbate reductase.

A2: Primers used for real-time RT-PCR.
